# Shielding of Lipid Nanoparticles for siRNA Delivery: Impact on Physicochemical Properties, Cytokine Induction, and Efficacy

**DOI:** 10.1038/mtna.2014.61

**Published:** 2014-11-18

**Authors:** Varun Kumar, June Qin, Yongfeng Jiang, Richard G Duncan, Benjamin Brigham, Shannon Fishman, Jayaprakash K Nair, Akin Akinc, Scott A Barros, Pia V Kasperkovitz

**Affiliations:** 1 Alnylam Pharmaceuticals, Cambridge, Massachusetts, USA

**Keywords:** delivery, immune response, nanomedicine, nanoparticles, polyethylene glycol, RNAi

## Abstract

Formulation of short interfering RNA (siRNA) into multicomponent lipid nanoparticles (LNP) is an effective strategy for hepatic delivery and therapeutic gene silencing. This study systematically evaluated the effect of polyethylene glycol (PEG) density on LNP physicochemical properties, innate immune response stimulation, and *in vivo* efficacy. Increased PEG density not only shielded LNP surface charge but also reduced hemolytic activity, suggesting the formation of a steric barrier. In addition, increasing the PEG density reduced LNP immunostimulatory potential as reflected in cytokine induction both *in vivo* and *in vitro*. Higher PEG density also hindered *in vivo* efficacy, presumably due to reduced association with apolipoprotein E (ApoE), a protein which serves as an endogenous targeting ligand to hepatocytes. This effect could be overcome by incorporating an exogenous targeting ligand into the highly shielded LNPs, thereby circumventing the requirement for ApoE association. Therefore, these studies provide useful information for the rational design of LNP-based siRNA delivery systems with an optimal safety and efficacy profile.

## Introduction

Recent progress in clinical development of RNA interference (RNAi) therapeutics has demonstrated that formulation of short interfering RNA (siRNA) into multi-component lipid nanoparticles (LNPs) is an effective strategy for liver delivery and silencing of therapeutically relevant gene targets.^1,2^ Careful design of various parameters is critical for optimizing pharmacokinetic and pharmacodynamic parameters while minimizing potential for adverse effects due to activation of the immune response. The innate immune system is the first line of defense against invading pathogens.^3,4^ It continuously monitors the body to discriminate between self and nonself molecular patterns using cellular pattern recognition receptors and soluble proteins such as complement factors. Detection of a foreign pattern results in an acute inflammatory response that is aimed at removal of the foreign substance and characterized by induction of proinflammatory mediators such as cytokines and chemokines. Conventional liposomes are known to interact with certain serum factors, including complement, which can cause an acute hypersensitivity reaction (also known as pseudoallergy) upon first exposure to the drug.^5^ In addition, complement activation can result in particle opsonization followed by rapid recognition and clearance by the mononuclear phagocyte system.^6^ Stealth nanocarriers, with a surface steric barrier of adsorbed/grafted hydrophilic polymer, were developed to improve circulation half-life^7,8,9^ by avoiding recognition by the mononuclear phagocyte system.^6,10,11,12^ Polyethylene glycol (PEG) has been used extensively as a stealth coating.^7,9,13^ The protection provided by PEG against protein binding and complement activation has been well-established,^14,15,16,17^ and both the PEG molecular weight and shielding density have been found to impact particle opsonization and subsequent clearance.^13,18,19^ For LNP-formulated RNAi therapeutics, the lipid vehicle and the siRNA payload both form potential targets for the innate immune system. Chemically unmodified siRNAs can induce proinflammatory cytokines such as IL-6, TNF-α, and IFN-α.^20,21^ Therefore, more recent clinical applications of RNAi typically employ siRNAs that contain chemical modification patterns that minimize immunostimulatory potential without interfering with gene silencing.^1,2,21,22,23^ However, even when using modified siRNA within LNPs, minor and transient elevations of serum cytokines can still sometimes be observed both preclinically and clinically,^24,25^ suggesting that the lipid components may contribute to the immunostimulatory potential of LNP-formulated siRNAs. To date, the study of the impact of PEG shielding in the context of LNP-mediated siRNA delivery has been limited^26^ and the effect on proinflammatory cytokine induction is still unclear. In the current study, we performed a systematic characterization of the effects of LNP PEG density on physicochemical properties, cytokine induction and delivery efficacy.

## Results

### Increasing the PEG density shields the surface charge of LNPs and reduces hemolytic activity

Various techniques for formulating LNPs have previously been reported and discussed.^27,28^ Self-assembly of key lipid components—ionizable lipid, neutral lipid, and stabilizing lipid—can be controlled to formulate LNPs with varying physiochemical properties. Inclusion of an ionizable lipid ensures efficient entrapment of anionic RNA during formulation under acidic conditions (pH < pKa of ionizable lipid, and hence positively charged lipid) while minimizing charge-mediated toxicity under physiological conditions (pH > pKa of ionizable lipid, and hence uncharged lipid). The ionizable lipid has also been hypothesized to help in the endosomal release of siRNA as the lipids are protonated under acidic endo/lysosomal conditions.^29^ The pKa value of the ionizable lipid can be tuned by changing the head group of the lipid.^30^ To evaluate the effect of PEG-lipid shielding on LNP surface charge, LNPs were formulated with varying levels of initial input PEG-lipid (based on lipid molar %): 1.5% (LNP1.5), 5% (LNP5), and 10% (LNP10). In contrast to the more conventional single-stage mixing formulation technique, we used a novel way of adding PEG-DMG at a later stage to preassembled particles of cholesterol, DSPC, DLin-MC3-DMA, and siRNA. This method allowed us to achieve the desirable surface density of PEG-DMG while still obtaining particles of similar final size (40–60 nm). The siRNA duplex used in these studies targeted murine blood coagulation Factor VII and was described previously.^31^ The final LNP PEG-lipid density was determined by measuring the lipid composition of LNPs separated from free PEG-lipid using size exclusion chromatography. LNPs are eluted in earlier void fractions compared to free PEG-lipid micelles. The eluted fractions corresponding to LNPs were collected and the lipid compositions were analyzed using high-performance liquid chromatography. The measured value of PEG-lipid composition of LNP1.5 was ~1.4–1.5% and LNP5 was ~4–5%. However, a lower value of 6–8% was observed for LNP10, which suggests a saturation limit of available surface area for 40–60 nm sized particles. We confirmed that the final particle size for LNP1.5, LNP5, and LNP10 was similar (see **Supplementary Table S1** for particle size, polydispersity index, and siRNA entrapment).

**Figure 1:**
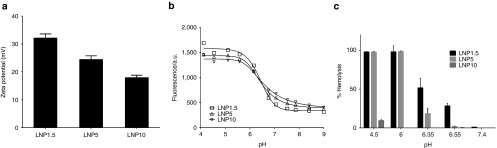
**Increasing the polyethylene glycol (PEG) density shields the surface charge of lipid nanoparticles (LNPs) and reduces hemolytic activity.** (**a**) Zeta potential for LNP1.5, LNP5, and LNP10. Zeta potential was measured at pH = 5.5 in RO/DI water. (**b**) 2-(p-toluidinyl) naphthalene-6-sulfonic acid, sodium salt fluorescence across a range of pH values for LNP1.5, LNP5, and LNP10. A characteristic fluorescent signal drop is attributed to pKa of the ionizable lipid, and an asymmetric five parameter fit was used to capture it. (**c**) Hemolytic activity across a range of pH values for LNP1.5, LNP5, or LNP10. The relative hemolysis compared to complete hemolysis (100%) induced by 2% Triton X-100 is shown.

To evaluate the effect of PEG density on the physicochemical properties of the particles, we assessed the LNP surface charge using multiple methods. Zeta potential measurement was performed at a pH of 5.5 (pH < pKa of the ionizable lipid) to ensure protonation of surface ionizable lipids and in deionized water diluent to eliminate charge shielding by negatively charged counterions, thus enabling detection of charge shielding as a function of PEG density. Shielding of the surface charge on LNPs by increasing the PEG density was observed as the zeta potential dropped from +32 mV (for LNP1.5) to +24 (for LNP5) and +18 mV (for LNP10), and is shown in **Figure 1a**. Additionally, the apparent surface charge was assessed by the 2-(p-toluidinyl) naphthalene-6-sulfonic acid, sodium salt (TNS) assay (**Figure 1b**) across a range of pH to establish the pKa trend for LNPs.^32^ This assay is based on the fluorescence enhancement of TNS molecules in the hydrophobic lipid domain, as they are electrostatically attracted to the positively charged particles. The absolute value of fluorescence intensity in the pH = 4–5 range, when ionizable lipids are positively charged, can be correlated with the particle surface charge (zeta potential): a higher intensity was observed for a relatively higher charged particle. A characteristic fluorescent signal drop was observed for each LNP with an increase in pH that is attributed to the pKa of the ionizable lipid. Another method that is useful for assessing the cationic charge on particles is the hemolytic assay, which is based on the electrostatic interaction and fusion of LNPs with anionic membranes of red blood cells (RBCs), resulting in lysis of the RBCs. The assay has been reported in the literature primarily as a screening tool for cationic lipids as it is a potential model for lipid fusogenicity with the endosomal membrane and subsequent release of active drugs.^33,34^ **Figure 1c** summarizes the hemolytic activity for LNPs as the surface charge is titrated by the change in pH. For LNP1.5, a very smooth hemolytic trend was observed with pH titration. At low pH, LNP1.5 produced complete hemolysis, which decreased as pH approached the pKa of the ionizable lipid. No hemolytic activity was observed at neutral pH. For LNP5, full hemolysis was observed up to pH = 6, which was then followed by a very steep drop in hemolytic activity as the pH approached the pKa of the lipid. Interestingly, in contrast to LNP1.5 and LNP5, LNP10 had a very low hemolytic activity across all pH conditions. These data suggest that the low hemolytic activity for LNP10, even at a lower pH = 4.5, can mainly be attributed to the steric PEG barrier in the LNP-RBC interaction, and not to the apparent surface charge of LNP10 (**Figure 1b**). Therefore, collectively, the data suggest that increasing LNP PEG density shields surface charge and inhibits membrane fusogenicity.

### Higher PEG density reduces LNP-mediated immunostimulation *in vivo*

**Figure 2:**
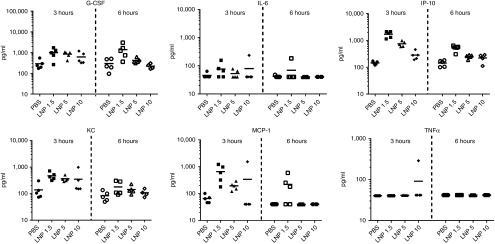
**Higher polyethylene glycol (PEG) density reduces lipid nanoparticles (LNP)-mediated immunostimulation *in vivo*.** Serum cytokine profile in CD-1 mice after intravenous administration of 10 mg/kg siRNA formulated in LNP1.5, LNP5, or LNP10 (5 animals per group, individual animals are shown with mean indicated by horizontal black bars). Serum was collected 3 and 6 hours postdose. For statistical analysis using factorial analysis of variance and Tukey's *post hoc* test, see **Supplementary Table S1**.

Next, we investigated the effect of increased PEG density on the potential of the LNPs to activate the innate immune system. Lipid composition, charge, and size are all known to affect particle interaction with the immune system.^6,35,36^ To evaluate the effect of PEG density on LNP immunostimulatory potential, we analyzed cytokine induction by LNP1.5, LNP5, and LNP10 in an *in vivo* model using the CD-1 outbred mouse strain (see **Figure 2**). As explained above, the siRNA used in these studies was chemically modified to minimize its contribution to the immunostimulatory potential of the LNP. Mice (five/group) were administered an exaggerated dose of 10 mg/kg siRNA formulated in LNP1.5, LNP5, or LNP10 or phosphate buffer saline as a control. Serum was collected at 3 and 6 hours postinjection for cytokine analysis. Sera were analyzed using a custom Milliplex MAP Mouse Cytokine/Chemokine Magnetic Bead Panel (Millipore, Bellerica, MA) and the analytes included were granulocyte colony stimulating factor (G-CSF), tumor necrosis factor (TNF)-α, interleukin (IL)-6, IP-10, keratinocyte-derived cytokine (KC), and monocyte chemotactic protein-1 (MCP-1) (**Figure 2**). Factorial analysis of variance was used to test the effect of analyte, time, treatment, and interactions on log-transformed cytokine level. Main effects were all significant (analyte, *P* < 0.001; time, *P* < 0.001; treatment, *P* < 0.001). All interactions were significant at *P* < 0.01. Tukey's *post hoc* tests assessed individual pairwise comparisons within analyte/time combinations (see **Supplementary Table S2** for a list of complete adjusted *P* values). The results clearly demonstrated that LNP1.5 induced higher overall cytokine levels than LNP5 and LNP10. This difference was particularly marked for IP-10, KC, and MCP-1 at 3 hours postdose and for G-CSF at 6 hours postdose.

### Higher PEG density reduces LNP-mediated immunostimulation *in vitro*

**Figure 3:**
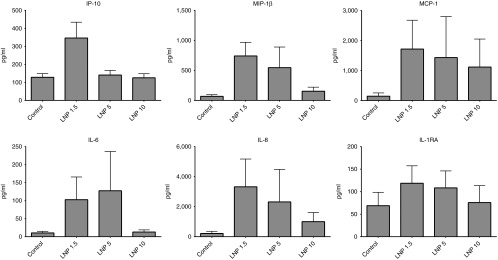
**Higher polyethylene glycol (PEG) density reduces LNP-mediated immunostimulation *in vitro*.** Human plasma cytokine profile after *in vitro* incubation of whole blood with 300 nmol/l siRNA formulated in lipid nanoparticles (LNP)1.5, LNP5, or LNP10 for 24 hours. A total of 13 cytokines were analyzed using blood from multiple donors (*n* = 4). Two-way analysis of variance examined the effect of treatment and analyte; Tukey's honestly significant difference pairwise comparisons showed significant cytokine elevations for LNP1.5 (*P* < 0.0001) and for LNP5 (*P* = 0.02) when compared to the medium alone control. In addition, cytokine levels were significantly higher for LNP1.5 compared to LNP10 (*P* = 0.002). The six individual analytes that best illustrate this trend are shown below (mean ± SEM for four blood donors). The other cytokines that were evaluated but showed either overall low expression or minor changes upon treatment were IL-1β, IL-10, IL-12, G-CSF, IFN-γ, MIP-1α, TNFα (data not shown).

As a next step, we applied a human whole blood cytokine assay to determine the impact of LNP PEG density on interaction with human immune cells (**Figure 3**). Whole blood from four healthy donors was treated with 300 nmol/l siRNA formulated in LNP1.5, LNP5, or LNP10 or a medium only control. Plasma was collected after 24 hours and analyzed using a custom Bio-Plex Pro Magnetic Bead Panel (BioRad Laboratories, Hercules, CA). Analytes included IL-1β, IL-1RA, IL-6, IL-8, IL-10, IL-12(p70), IP-10, G-CSF, interferon (IFN)-γ, MCP-1, MIP-1α, MIP-1β, and TNF-α. Two-way analysis of variance was used to test the effect of treatment and analyte; Tukey's honestly significant difference pairwise comparisons showed significant cytokine elevations for LNP1.5 (*P* = 0.00001) and for LNP5 (*P* = 0.02) when compared to the medium only control. In addition, cytokine levels were significantly higher for LNP1.5 compared to LNP10 (*P* = 0.002). The six individual analytes that best illustrate this trend are shown in **Figure 3**. The other cytokines that were evaluated showed either overall low expression or only minor changes upon treatment (data not shown). These data demonstrate that increasing PEG density reduces cytokine induction in a human model system and further support the concept that increasing the PEG density may be a valuable strategy to reduce LNP acute immunostimulatory potential and cytokine induction.

### Impact of PEG density on *in vivo* hepatic delivery by ApoE- and ASGPR-mediated mechanisms

**Figure 4:**
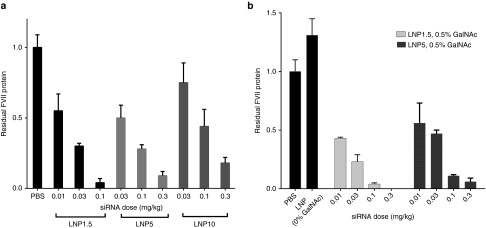
**Effect of high polyethylene glycol (PEG) shielding on efficacy of FVII silencing after i.v. targeting of hepatocytes by ApoE- and ASGPR-mediated routes.** (**a**) Dose-response of siRNA-mediated Factor VII silencing after i.v. delivery of lipid nanoparticles (LNP)1.5, LNP5, or LNP10. Serum Factor VII protein levels were measured in C57BL/6 mice (*n* = 4) 48 hours post-i.v. administration. (**b**) Silencing is abrogated in ApoE^−/−^ mice (black bars). Surface addition of targeting ligand (GalNAc) to LNP1.5 (with 0.5% GalNAc-PEG-DSG) and LNP5 (with 0.5% GalNAc-PEG-DSG) restores silencing (light and dark grey bars). Serum Factor VII protein levels were measured in ApoE^−/−^ mice (*n* = 3) 48 hours post-i.v. administration.

Finally, we studied the impact of PEG density on the efficacy of LNP-mediated siRNA delivery using a murine *in vivo* model system described previously.^31^ LNP1.5, LNP5, or LNP10 were formulated using the same siRNA used above that targets mouse blood coagulation factor VII (FVII).^31^ Because FVII is synthesized in the liver and released into the blood, serum protein levels of FVII reflect the level of siRNA-mediated gene silencing. LNPs were administered i.v. at three doses and serum was collected 48 hours postadministration and analyzed for FVII protein levels (**Figure 4a**). Of the three formulations, LNP1.5 was clearly the most efficacious. With increased PEG density, a trend in reduced efficacy was observed, and higher doses were required to achieve efficient silencing. The observed reduction in efficacy with increased PEG density may be attributable to the mechanism of LNP delivery to hepatocytes *in vivo*. It has previously been shown that the targeting of LNPs to hepatocytes *in vivo* is mediated by apolipoprotein E (ApoE), requiring the association of serum ApoE with the LNP particle surface for functional delivery.^31^ When we performed a series of studies in apoE^−/−^ mice, we found that preincubation of LNPs with recombinant ApoE before injection rescued the activity of LNP1.5, whereas no improvement in efficacy was observed for LNP5 (see **Supplementary Figure S1**). In addition, *in vitro* binding studies showed reduced association of ApoE to LNP5 compared to LNP1.5 (see **Supplementary Figure S2**). As our results suggest that an endogenous ApoE-mediated delivery mechanism is inhibited by increased LNP PEG density, we explored whether this limitation could be circumvented by utilizing an ApoE-independent delivery strategy. Specifically, Akinc *et al*.^31^ have previously shown that the activity of LNPs can be rescued in apoE^−/−^ mice by using an exogenous targeting ligand containing a multivalent N-acetylgalactosamine (GalNAc)-cluster, which binds to the asialoglycoprotein receptor on hepatocytes. Therefore, GalNAc-PEG-DSG (0.5 mol%, based on total lipids) was incorporated into LNPs with high or low PEG density. **Figure 4b** compares the potency of GalNAc-LNPs with low PEG (0.5% GalNAc-PEG-DSG + 1% PEG-DMG) and high PEG (0.5% GalNAc-PEG-DSG + 4.5% PEG-DMG) density. These data show very similar, dose-dependent efficacy for both LNP1.5 and LNP5, indicating that limitations in efficacy due to increased PEG shielding can successfully be overcome by incorporating exogenous targeting ligands.

## Discussion

The findings described in this article emphasize the importance of the PEG-lipid shield on the physicochemical and functional properties of LNPs. We modulated LNP PEG-lipid density by utilizing a two-stage formulation process whereby additional PEG-lipid could be titrated on the surface of preformed LNPs of a defined particle size range, resulting in similarly sized particles for LNP1.5, LNP5, and LNP10. Previous data by Bao *et al*.^26^ demonstrated that increasing the PEG-C-DMA to lipid ratio did not alter the hepatic distribution of LNPs but reduced hemolytic activity and negatively affected gene silencing efficacy. However, the LNPs used in these studies were assembled using the conventional formulation technique of mixing all the components at a single stage, which results in smaller particles at higher PEG-DMA concentrations due to faster stabilization kinetics. Specifically, the LNPs described by Bao *et al*. varied in final particle size from 103 nm for 1% PEG-DMA to 65 nm for 10% PEG-DMA. In contrast, our formulation method produced similarly sized particles for LNP1.5 (58 nm) and LNP10 (46 nm) (also see **Supplementary Table S1**). Keeping the final particle size consistent is critical for evaluation of the functional properties of the LNPs since particle size may impact pharmacokinetics, biodistribution, and cellular uptake, which can in turn affect both gene silencing efficacy and immunostimulation. Our data demonstrate that increasing the LNP PEG density shield from 1.5 to 5% or 10% resulted in a concomitant decrease in particle surface charge and hemolytic activity. These data are in agreement with the previously observed reduction in hemolytic activity with increased PEG-C-DMA to lipid ratio which was assumed to be due to charge shielding.^26^ However, our detailed analysis of particle surface charge titration with pH combined with hemolytic activity indicated that the physical steric barrier effect provided by high PEG density appeared to be more significant than the charge shielding effect in inhibiting membrane fusion and disruption; LNP10 was observed to have a very low hemolytic activity as compared to LNP1.5, even at low pH where LNP10 was found to have positive surface charge (**Figure 1b**,**c**).

Interaction of LNP particles with cellular and humoral components of the immune system is also affected by surface charge and composition.^6,35,36^ In a study using gold nanoparticles, size and surface chemistry were found to control particle serum protein binding and subsequent uptake by macrophages.^14^ Increasing PEG grafting density decreased the total serum protein adsorption, including the major opsonin Complement protein 3 (C3), and reduced particle macrophage uptake.^14^ Our study showed that increased PEG shielding reduced overall cytokine production both in a murine *in vivo* model and a human *in vitro* model. All the cytokines that were measured can be produced by a variety of cells, including phagocytes, endothelial cells and hepatocytes. IL-6 is an acute phase response protein that plays a role in fever induction while G-CSF promotes granulocyte maturation. Both were also observed in clinical application at higher doses.^24^ IP-10 is a chemokine that is induced as part of the antiviral IFN response and has also been observed in preclinical and clinical studies at higher doses.^24^ MCP-1 and KC are pleiotropic chemokines that attract phagocytes such as monocytes and neutrophils. Interestingly, TNFα induction was not observed, which is in agreement with previous findings demonstrating that a TNF-α response to unmodified siRNA can be successfully abrogated through chemical modification of the siRNA.^23,37^ Because the liver is the main organ of drug distribution,^24,26^ it is likely that the liver phagocytes (Kupffer cells) and potentially the hepatocytes contribute to the observed immune response.^26,38^ Taken together, our data suggest that increasing PEG density is a valuable strategy to reduce undesirable interactions between the particle and the immune system.

The effect of PEG density on LNP hepatic delivery and RNAi efficacy appears to be dependent on the mode of hepatic targeting. Increased PEG density negatively impacted the efficacy of LNPs, likely by inhibiting the endogenous ApoE-mediated targeting mechanism. Our observations further support the hypothesis that a high PEG lipid shield has the potential to reduce the interaction of serum ApoE with LNPs. In fact, the LNP systems developed for hepatic siRNA delivery have been designed to have minimal PEG shielding that is rapidly de-shielded *in vivo*.^39^ The rate of deshielding of the LNP is determined by the exchangeability of the PEG-lipid into serum lipoproteins, which is controlled by dialkyl chain length of the lipid to which the PEG is conjugated.^40^ Specifically, PEG-lipids with a dialkyl chain length of 14 carbons (*e.g.*, PEG-DMG) are rapidly exchanged out of the LNP, whereas those with a dialkyl chain length of 18 carbons (*e.g.*, PEG-DSG) are stably associated with the LNP. However, our data show that this apparent limitation of increased PEG shielding may be successfully overcome by using an ApoE-independent delivery strategy. Incorporation of an exogenous targeting ligand containing a multivalent N-acetylgalactosamine (GalNAc)-cluster, which binds to the asialoglycoprotein receptor on hepatocytes, successfully circumvented the requirement for ApoE association.

In summary, increasing PEG density shields LNP surface charge and reduces immunostimulatory potential, which may contribute to improved safety and reduced clearance by the mononuclear phagocyte system. The negative impact of increased PEG density of LNP efficacy may be successfully overcome by incorporating exogenous targeting ligands, thereby circumventing the requirement for ApoE association. Therefore, these studies provide useful information for the rational design of LNP-based siRNA delivery systems with an optimal safety and efficacy profile.

## Materials and Methods

*Lipid nanoparticle formulation.* LNPs were formulated using the rapid precipitation method. Organic lipid solution (with lipid composition of heptatriaconta-6,9,28,31-tetraen-19-yl4-(dimethylamino)butanoate (DLin-MC3-DMA)^30^: distearoylphosphatidylcholine (DSPC): cholesterol in a molar ratio of 51:10:39 in ethanol) was mixed with aqueous siRNA solution (siRNA dissolved in 25 mmol/l acetate buffer, pH = 4) via a T-junction. The total lipid to siRNA ratio was 9.75 (by wt.), and the siRNA concentration was 0.6 mg/ml in the solution after mixing. Polyethylene glycol (2K)-dimyristolglycerol (PEG-DMG) solution (concentration was adjusted to give desired amount of PEG density on LNPs) in ethanol was added to the mixed solution via a T-junction to provide steric stabilization to formed nanoparticles. Nanoparticle solution was instantly transferred for overnight dialysis at room temperature against 1× phosphate buffer saline to remove ethanol. Zetasizer (Malvern instruments, Westborough, MA) was used to determine the particle size (expressed as the intensity weighted diameter) and zeta potential. Zeta potential was measured on particles after suspending them in deionized water at pH = 5.

*Hemolysis assay.* Whole blood from C57BL/6 mice was collected into EDTA containing vacutainer tubes (BD, Franklin Lakes, NJ). Whole blood was centrifuged to harvest RBC and washed three times with 150 mmol/l saline. RBC were then suspended into 100 mmol/l phosphate buffer at various pH. For the hemolysis assay, RBC were diluted 1:10 in desired pH maintained phosphate buffer. 0.3 ml of the diluted RBC solution was mixed with 0.1 ml LNP (at 0.4 mg/ml concentration, based on siRNA) or with triton X-100 (as positive control for full hemolysis), and incubated at 37 °C for 20 minutes. The mixed solution was then centrifuged and the supernatant was collected for heme-absorbance analysis at 541 nm.

*TNS assay.* This assay was adapted from Heyes *et al*.^32^ Briefly, MES and HEPES were used to make buffers in the pH range of 4–9. The assay was performed in 96-well plates and per well, 157 µl of buffer with varying pH was added and mixed with 10 µl of LNP (at 0.2 mmol/l DLin-MC3-DMA concentration) and 16.7 µl of 0.01 mmol/l TNS. The fluorescence was measured at *λ*_em_ = 445 nm with *λ*_ex_ =321 nm.

*Human whole blood cytokine assay.* Whole blood from four anonymous, healthy donors was collected in Sodium Heparin Vacutainer tubes (BD), diluted 1:1 (v:v) in 0.9% Saline (Baxter, Deerfield, IL), and plated in 96-well flat bottom tissue culture plates at 180 μl/well. Whole blood was treated with Opti-MEM (Life Technologies, Carlsbad, CA) or 300 nmol/l siRNA formulated in LNP1.5, LNP5, or LNP10. Treated whole blood was incubated at 37 °C, 5% CO2 for 24 hours after which plasma was collected and stored at −80 °C until analysis. Cytokine levels were measured using a custom Bio-Plex Pro Magnetic Cytokine Assay (BioRad Laboratories), analytes included: IL-1β, IL-1RA, IL-6, IL-8, IL-10, IL-12(p70), IP-10, G-CSF, IFN-γ, MCP-1, MIP-1α, MIP-1β, and TNF-α.

*Mouse in vivo cytokine assay.* All animal procedures were conducted in accordance with the protocol approved by the Alnylam Institutional Animal Care and Use Committee. Male CD1 mice (age: 7–8 weeks, weight: 25–35 g) were obtained from Charles River Laboratories (Wilmington, MA). siRNA formulated in LNP1.5, LNP5, or LNP10 was diluted with sterile phosphate-buffered saline (Gibco, Carlsbad, CA) and animals were administered a total dose of 10 mg/kg (based on siRNA) or saline control via intravenous bolus injection using a 1 ml syringe and 27G needle at a dose volume of 10 ml/kg. After 4 hours, blood was collected via cardiac puncture into serum separator tubes (BD) and serum was stored at −80 °C until analysis. Cytokine analysis was done using a custom Milliplex MAP Mouse Cytokine/Chemokine Magnetic Bead Panel (Millipore) and the analytes included were G-CSF, TNF, IL-6, IP-10, KC, and MCP-1.

**SUPPLEMENTARY MATERIAL** **Figure S1.** Preincubation with ApoE rescues efficacy of LNP1.5 but not LNP5 in apoE -/- mice. **Figure S2.** In vitro ApoE/LNP binding assay. **Table S1.** Particle characterization details. **Table S2.** Adjusted p-values for Figure 2.
