# Supplementary material for: Shielding of Lipid Nanoparticles for siRNA Delivery: Impact on Physicochemical Properties, Cytokine Induction, and Efficacy
Source: Mol Ther Nucleic Acids. 2014 Nov 18;3(11):e210–. doi: 10.1038/mtna.2014.61 (PMC4459547; doi:10.1038/mtna.2014.61)
Supplement: Supplementary Information [file mtna201461x1.pdf]

SUPPORTING INFORMATION

Supplementary Figure 1: Preincubation with ApoE rescues efficacy of LNP1.5 but not LNP5 in apoE -/- mice

LNP1.5 or LNP5 were pre-incubated with recombinant ApoE (1:1 siRNA:apoE by wt; 8HIS-rhApoE was purified in house from E. coli) for 1 h at room temperature and then administered i.v. in apoE -/- mice at 0.1 mg/kg (based on entrapped siRNA conc.). FVII protein level was measured 48 h post dose.

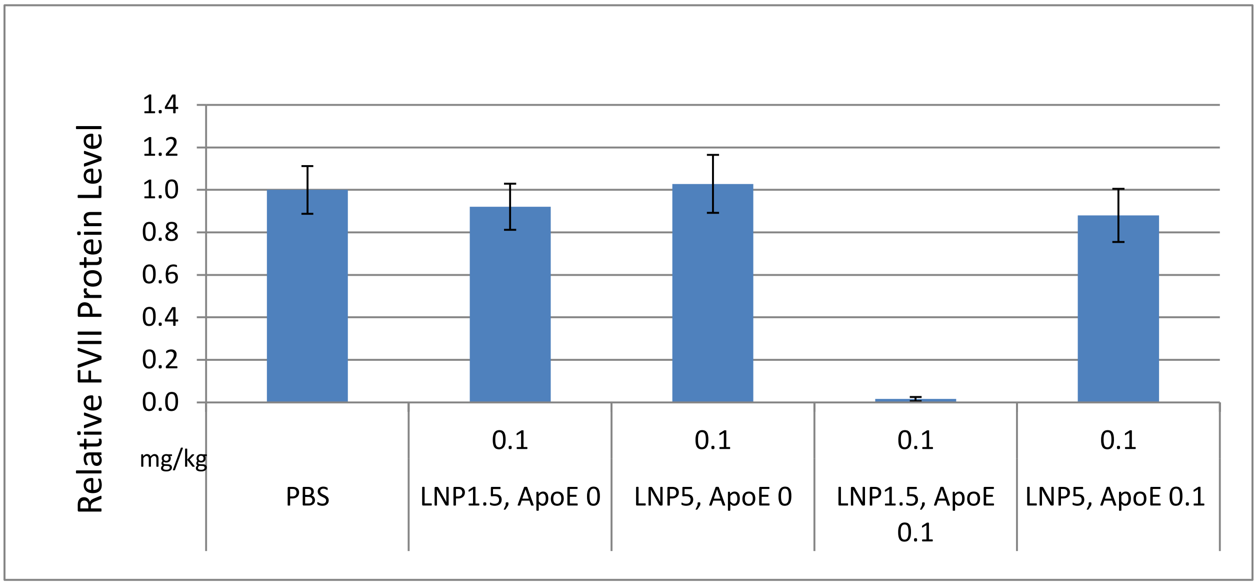

Supplementary Figure 2: *In vitro* ApoE/LNP binding assay

To determine ApoE/LNP interaction, 4 ng ApoE -Alexa647 (8HIS-rhApoE was purified in house from E. coli and Alexa647 purchased from Life technologies) was incubated with 38 ng LNP (based on entrapped siRNA concentration) in 1x PBS prior to elution over a Superose 6 PC 3.2/30 column (GE) in 1X PBS. The fractions (25 ul) through the column were collected and read on an M2 SpectraMax (Molecular Devices). The fluorescence emission data were collected at 670 nm using the excitation wavelength of 635 nm.

(a) Fluorescence data from the size exclusion column comparing the apoE-Alexa647 binding to LNP1.5 and LNP5 after 7 mins of apoE-Alexa647 / LNP incubation. The fluorescence signal in the fraction b1-b10 is from the LNP-bound apoE-Alexa647.

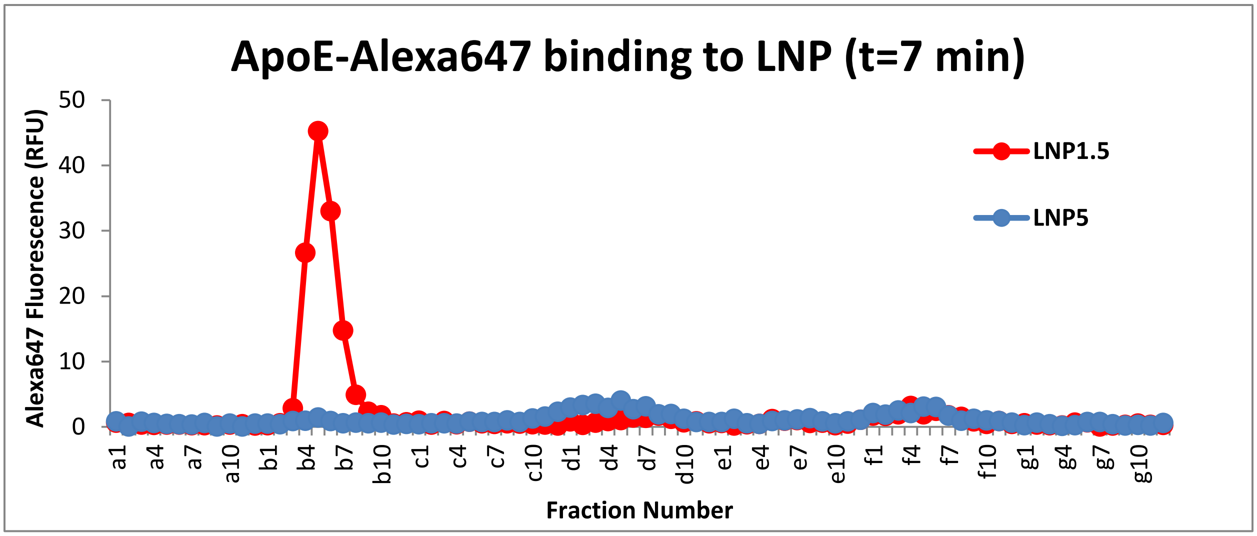

(b) ApoE-Alexa647 binding kinetics for LNP1.5 and LNP5 were studied by varying the incubation time of ApoE-Alexa647 with LNP. LNP1.5 was found to have instant binding to apoE-Alexa647, whereas LNP5 was found to have a very low binding affinity.

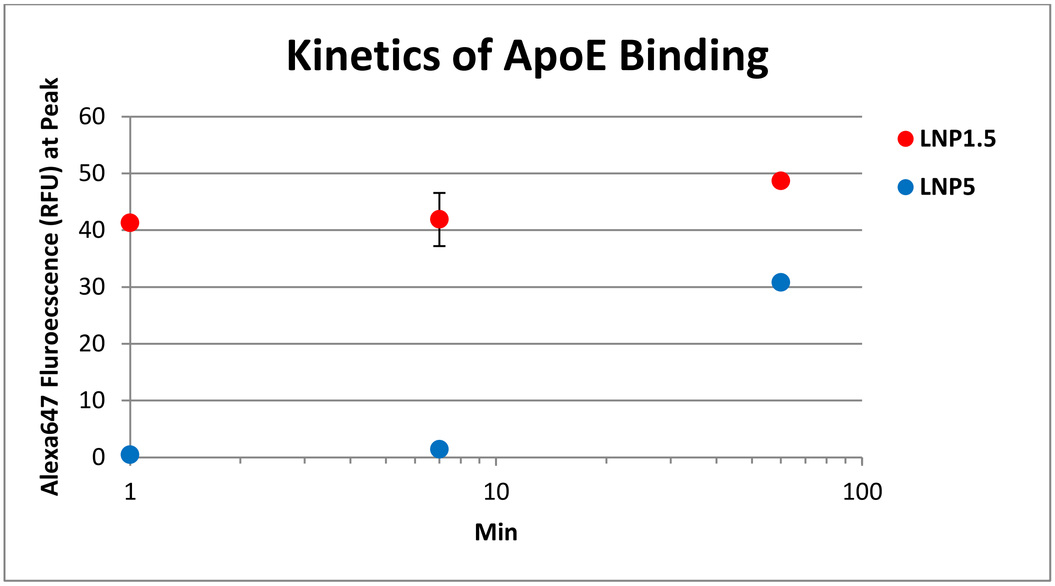

Supplementary Table 1: Particle characterization details

All dose calculations were based on entrapped siRNA concentration.

	Particle size (nm)	PDI	siRNA entrapment efficiency (%)
LNP1.5	58	0.088	96
LNP5	47	0.127	81
LNP10	46	0.139	82

Supplementary Table 2: Adjusted p-values for Figure 2

Least square means estimates of differences in analyte levels under each treatment vs. PBS control, at 3 and 6 hour timepoints. Estimates derived from ANOVA model; P values for pairwise comparisons according to Tukey's post hoc tests.

Analyte	Treatment	Timepoint	Difference *vs.* PBS	P-value *vs.* PBS
G-CSF	LNP1.5	3 hr	0.501142	0.1982
G-CSF	LNP5	3 hr	0.475255	0.3062
G-CSF	LNP10	3 hr	0.306124	0.9902
G-CSF	LNP1.5	6 hr	0.644772	0.0067
G-CSF	LNP5	6 hr	0.212687	1
G-CSF	LNP10	6 hr	-0.059909	1
IL-6	LNP1.5	3 hr	0.178498	1
IL-6	LNP5	3 hr	0.055722	1
IL-6	LNP10	3 hr	0.108962	1
IL-6	LNP1.5	6 hr	0.117735	1
IL-6	LNP5	6 hr	-0.014705	1
IL-6	LNP10	6 hr	-0.014705	1
IP-10	LNP1.5	3 hr	1.058799	<.0001
IP-10	LNP5	3 hr	0.705838	0.0011
IP-10	LNP10	3 hr	0.274785	0.9987
IP-10	LNP1.5	6 hr	0.560255	0.0587
IP-10	LNP5	6 hr	0.252131	0.9998
IP-10	LNP10	6 hr	0.175602	1
KC	LNP1.5	3 hr	0.577418	0.0392
KC	LNP5	3 hr	0.472336	0.3203
KC	LNP10	3 hr	0.333524	0.9626
KC	LNP1.5	6 hr	0.302459	0.992
KC	LNP5	6 hr	0.244405	0.9999
KC	LNP10	6 hr	0.125703	1
MCP-1	LNP1.5	3 hr	0.925123	<.0001
MCP-1	LNP5	3 hr	0.470999	0.3269
MCP-1	LNP10	3 hr	0.122065	1
MCP-1	LNP1.5	6 hr	0.523625	0.1291
MCP-1	LNP5	6 hr	0	1
MCP-1	LNP10	6 hr	0	1
TNFa	LNP1.5	3 hr	0	1
TNFa	LNP5	3 hr	0.012799	1
TNFa	LNP10	3 hr	0.184216	1

TNFa	LNP1.5	6 hr	0	1
TNFa	LNP5	6 hr	0	1
TNFa	LNP10	6 hr	0	1
